# DIGAP - a Database of Improved Gene Annotation for Phytopathogens

**DOI:** 10.1186/1471-2164-11-54

**Published:** 2010-01-21

**Authors:** Na Gao, Ling-Ling Chen, Hong-Fang Ji, Wei Wang, Ji-Wei Chang, Bei Gao, Lin Zhang, Shi-Cui Zhang, Hong-Yu Zhang

**Affiliations:** 1 National Key Laboratory of Crop Genetic Improvement, College of Life Science and Technology, Huazhong Agricultural University, Wuhan 430070, PR China; 2 Shandong Provincial Research Center for Bioinformatic Engineering and Technique, Center for Advanced Study, Shandong University of Technology, Zibo 255049, PR China; 3 Department of Marine Biology, Ocean University of China, Qingdao 266003, PR China

## Abstract

**Background:**

Bacterial plant pathogens are very harmful to their host plants, which can cause devastating agricultural losses in the world. With the development of microbial genome sequencing, many strains of phytopathogens have been sequenced. However, some misannotations exist in these phytopathogen genomes. Our objective is to improve these annotations and store them in a central database DIGAP.

**Description:**

DIGAP includes the following improved information on phytopathogen genomes. (i) All the 'hypothetical proteins' were checked, and non-coding ORFs recognized by the Z curve method were removed. (ii) The translation initiation sites (TISs) of 20% ~ 25% of all the protein-coding genes have been corrected based on the NCBI RefSeq, ProTISA database and an *ab initio *program, GS-Finder. (iii) Potential functions of about 10% 'hypothetical proteins' have been predicted using sequence alignment tools. (iv) Two theoretical gene expression indices, the codon adaptation index (CAI) and the *E*(*g*) index, were calculated to predict the gene expression levels. (v) Potential agricultural bactericide targets and their homology-modeled 3D structures are provided in the database, which is of significance for agricultural antibiotic discovery.

**Conclusion:**

The results in DIGAP provide useful information for understanding the pathogenetic mechanisms of phytopathogens and for finding agricultural bactericides. DIGAP is freely available at http://ibi.hzau.edu.cn/digap/.

## Background

Plant pathogenic bacteria are very harmful to their host plants, which can cause devastating agricultural losses in the world. The progress in bacterial genome sequencing project has enabled a better understanding of plant pathogens at the molecular level. Up to the middle of 2009, 28 strains of bacterial phytopathogen genomes have been sequenced, whose names and general annotation information are listed in Table 1. The availability of these phytopathogen genomes provides an unprecedented opportunity for the research of lifestyle and pathogenicity of plant pathogens as well as agricultural bactericide discovery.

**Table 1:** General annotation information of the 28 plant pathogens

Species ^a^	Abbreviation	RefSeq	Genomic Length (bp)	G+C content (%)	Annotated ORFs in RefSeq
*Acidovorax avenae *subsp. *citrulli *AAC00-1	*Aac*	NC_008752	5,352,772	68.02	4709
*Agrobacterium tumefaciens *str. C58	*At*58	NC_003062	2,841,580	59.38	2765
*Agrobacterium vitis S4*	*Av*4	NC_011989	4,009,526	57.60	4288
*Aster yellows witches'-broom phytoplasma *strain AY-WB	*Ayw*	NC_007716	706,595	26.89	671
*Clavibacter michiganensis *subsp. *michiganensis *NCPPB 382	*Cmm*	NC_009480	3,297,891	72.66	2984
*Clavibacter michiganensis *subsp. *sepedonicus *ATCC 33113	*Cms*	NC_010407	3,258,645	72.60	2941
*Candidatus Phytoplasma australiense*	*Cpa*	NC_010544	879,959	27.40	684
*Candidatus Phytoplasma mali*	*Cpm*	NC_011047	601,943	21.40	479
*Erwinia carotovora *subsp. *atroseptica *SCRI1043	*Eca*	NC_004547	5,064,019	50.97	4472
*Leifsonia xyli *subsp. *xyli *str. CTCB07	*Lxx*	NC_006087	2,584,158	67.68	2030
*Mesoplasma florum *L1	*MfL*	NC_006055	793,224	27.02	682
*Onion yellows phytoplasma *OY-M	*Oyp*	NC_005303	860,631	27.74	754
*Pseudomonas syringae *pv. *phaseolicola *1448A	*Psp*	NC_005773	5,928,787	58.02	4985
*Pseudomonas syringae *pv. *syringae *B728a	*Pss*	NC_007005	6,093,698	59.23	5089
*Pseudomonas syringae *pv. tomato str. DC3000	*Pst*	NC_004578	6,397,123	58.40	5476
*Ralstonia solanacearum *GMI1000	*Rs*1000	NC_003295	3,716,416	67.04	3438
*Xanthomonas axonopodis *pv. *citri *str. 306	*Xac*	NC_003919	5,175,554	64.77	4312
*Xanthomonas campestris *pv. *campestris *str. 8004	*Xcc*8004	NC_007086	5,148,708	64.96	4273
*Xanthomonas campestris *pv. *campestris *str. ATCC 33913	*Xcc*33913	NC_003902	5,076,188	65.07	4181
*Xanthomonas campestris *pv. *campestris *str. B100	*Xcc*100	NC_010688	5,079,002	65.00	4467
*Xanthomonas campestris *pv. *vesicatoria *str. 85-10	*Xcv*	NC_007508	5,178,466	64.75	4487
*Xanthomonas oryzae *pv. *oryzae *MAFF 311018	*Xoo*311018	NC_007705	4,940,217	63.70	4372
*Xanthomonas oryzae *pv. *oryzae *KACC10331	*Xoo*10331	NC_006834	4,941,439	63.69	4144
*Xanthomonas oryzae *pv. *oryzae *PXO99A	*Xoo*99A	NC_010717	5,240,075	63.60	4988
*Xylella fastidiosa *M12	*XfM*12	NC_010513	2,475,130	51.90	2104
*Xylella fastidiosa *M23	*XfM*23	NC_010577	2,535,690	51.80	2161
*Xylella fastidiosa *9a5c	*Xf*9a5c	NC_002488	2,679,306	52.67	2766
*Xylella fastidiosa Temecula1*	*XfT*	NC_004556	2,519,802	51.78	2034

^a ^For *Agrobacterium tumefaciens *str. C58, *Ralstonia solanacearum *GMI1000, only the largest chromosome are considered.

However, due to the absence of abundant experimental information, many misannotations still exist in the sequenced bacterial genomes, especially in GC-rich genomes [1-6]. Firstly, many bacterial genomes have false-positive gene identification, *i.e.*, some open-reading frames (ORFs) are incorrectly predicted as protein-coding genes; most of them are short ORFs (<150 bp) without functional information [1-3]. Secondly, many annotated genes have wrong translation initiation sites (TISs). It is indicated that up to 60% of the annotated genes in 143 prokaryotic genomes have wrong TISs in GenBank [7] or RefSeq [8], especially in GC-rich genomes [1]. Thirdly, a large number of function-unknown 'hypothetical proteins' are annotated in public databases, which account for 30% ~ 50% in different genomes [5,6]. These problems are even more serious in phytopathogen genomes because most of them are GC-rich (>50%). Here, we have constructed DIGAP to correct some mistakes and provide improved annotations for these plant pathogens.

## Construction and content

### Construction

**Figure 1:**
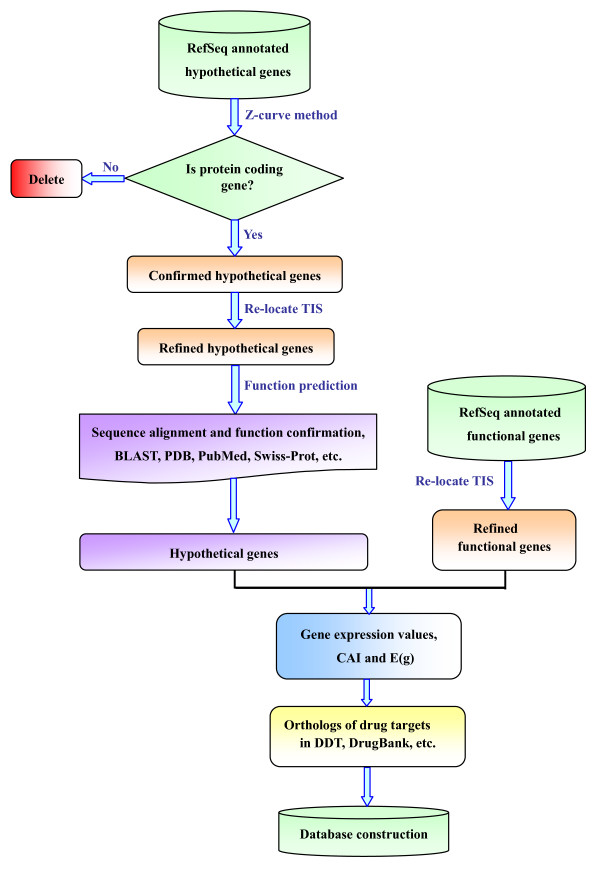
**Flowchart depicting the strategy of refined annotation for 28 plant pathogens**.

The construction of DIGAP was based on the LAMP platform, *i.e.*, an open source operation system Linux http://www.linux.org/, a stable web sever Apache http://www.apache.org, a fast database management system MySQL http://www.mysql.com and a powerful web scripting language PHP/Perl http://www.php.net, http://www.perl.org/. All the phytopathogen genomes were downloaded from NCBI RefSeq [8], release 33. The flowchart of the database construction is illustrated in Figure 1. Briefly, it contains the following steps.

### Content

#### Finding non-coding ORFs from annotated 'hypothetical ORFs'

The method adopted here was based on the Z curve of DNA sequence [9], which had been successfully applied to find genes in prokaryotic and some eukaryotic genomes [3,10-12]. In the present analysis, 21 variables are adopted, which include 9 phase-dependent single nucleotides and 12 phase-independent di-nucleotides. For details see [Additional file 1].

#### Relocating translation initiation sites

ProTISA is a recently constructed database, which provides experimentally confirmed and theoretically refined TISs for hundreds of prokaryotic genomes [13]. In addition, an *ab initio *TIS identification program GS-Finder [14] was employed to refine TISs in these plant pathogens. Joint-jury method was used to make the final decision. If two of the three systems (RefSeq, ProTISA and GS-Finder) had the same TIS, then it was predicted to be the true TIS. ProTISA is a comprehensive resource, which contained conserved domain confirmed (CDC) and high similarity confirmed (HSC) information for TISs [13]. Therefore, if the three systems predicted different TISs, the site provided by ProTISA was adopted. Five phytopathogen genomes *Av*4, *Cms*, *Cpa*, *Xcc*100 and *Xoo*99A were not contained in ProTISA, therefore only GS-Finder was used to relocate TISs for the five genomes.

#### Predicting hypothetical protein functions with sequence alignment

After removing the non-coding ORFs and correcting many TISs, the third step was to predict functions for the 'hypothetical proteins'. The sequence alignment tool BLAST [15] was used to search public non-redundant databases. Function was predicted to a 'hypothetical protein' if the aligned homologs had definite function which occurred more than five times with sequence alignment coverage >60%, sequence identity ≥40% and E value <1e-10. Then the predicted functions were searched in NCBI PubMed [16], Swiss-Prot [17] and PDB [18] to find experimentally characterized homologs. If a 'hypothetical protein' had PDB (or Swiss-Prot) homologs with the same function as predicted by sequence alignment, then the function of the 'hypothetical protein' and its PDB (or Swiss-Prot entry with evidence at protein level) homolog was listed in DIGAP.

#### Predicting gene expression levels

Codon adaptation index (CAI) and *E*(*g*) are theoretical indices which were used to predict gene expression levels in prokaryotic genomes [19,20]. To some extent the expression level of a gene can indicate the importance of its function. Some highly expressed genes are potential antibiotic targets in plant protection. Detailed methods to calculate CAI and *E*(*g*) values are listed in [Additional file 2]. The predicted highly expressed genes were marked with '*' in DIGAP.

#### Predicting potential bactericide targets and modeling their 3D structures

So far, hundreds of proteins and nucleic acids have been explored as therapeutic antibacterial targets in human and animals. Some databases, such as TTD [21] and DrugBank [22], have been constructed to provide information for the known targets in human and animal species. However, no such information is available for bacterial plant pathogens up to now. So we searched the orthologs of antibacterial targets in TTD and DrugBank, and listed all the potential bactericide targets in DIGAP. For each potential target, the protein sequence from a representative phytopathogen was selected, and homology modeling was employed to construct its 3D structure. First, similarity search was performed using BLAST against PDB to acquire the template. If there were multiple structural candidates in PDB for a certain protein, the one with inhibitor and the highest resolution was selected. Then, the 3D structure was constructed by employing the homology modeling module of Insight II software. Subsequently, molecular dynamics equilibration was performed to refine the obtained 3D structures with the consistent-valence force field (CVFF) on a SGI Origin 350 server. The models were minimized by 1000 conjugate gradient steps for equilibration, heated from 2 K to 300 K during 35 psec at temperature increment of 50 K per 5 psec, then the constant temperature and pressure algorithm was applied at 300 K for 200 psec. The velocity verlet integrator was used with an integration step of 2 fsec. Finally, the feasibility of modeled structures was evaluated by Verify3D to ensure that all the predicted structures had an acceptable 3D-1D self-compatibility score.

## Utility and discussion

**Figure 2:**
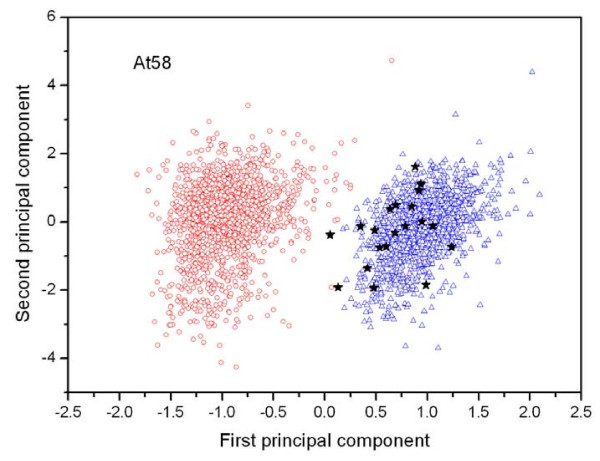
**The distribution of points on the principal plane spanned by the first (*x*) and second (*y*) principal axes using the principal component analysis (PCA) in *At*58**. The red circles represent the function-known genes, the blue triangles represent the corresponding negative samples and the black stars denote the recognized non-coding ORFs. The first and second principal axes account for 33.96% and 14.98% of the total inertia of the 21-dimensional space, respectively. It is clear that most of the identified non-coding ORFs distribute far from the core of open circles, and close to the core of open triangles, which implies that the recognized non-coding ORFs are very unlikely to encode proteins.

General results of the improved annotations are listed in Table 2. Firstly, all the "hypothetical proteins" in the original RefSeq annotation are re-analyzed by using the Z curve method [9]. About 1% ~ 3% of the 'hypothetical proteins' were recognized as non-coding ORFs in each phytopathogen genome, and are listed in the second column of Table 2. Differences between coding and non-coding sequences (positive and negative samples) can be intuitively viewed from principle component analysis (PCA). Figure 2 shows the distribution of points on the principal plane spanned by the first two principal components for *At*58. The red circles denote the function-known genes, and the blue triangles denote the corresponding shuffled sequences. The recognized non-coding ORFs are represented by black stars, which distribute far from the core of the function-known genes, and close to random sequences. Figures for other plant pathogens are in the 'documents' section of the website http://ibi.hzau.edu.cn/digap/document.php?page=3. The average length of recognized non-coding ORFs is much shorter than that of the function-known genes (Table V in 'statistics' section of the website, http://ibi.hzau.edu.cn/digap/statistics.php#5). All the evidence supports that the recognized non-coding ORFs are very unlikely to encode proteins. Protein identification (PID) numbers for these non-coding ORFs are listed in Table IV in the 'statistics' section of the website http://ibi.hzau.edu.cn/digap/statistics.php#4.

**Table 2:** Refined information of the 28 plant pathogens

Species ^a^	Number of non-coding ORFs	Number (percentage) of refined TISs	Number (percentage) of HPs assigned with functions ^b^	Number (percentage) of PHX genes ^c^	Number of potential drug targets
*Aac*	15	699 (14.9%)	105 (9.1%)	327 (7.0%)	35
*At*58	20	640 (23.3%)	233 (23.0%)	210 (7.7%)	39
*Av*4	7	1171 (27.4%)	437 (33.9%)	76 (1.8%)	45
*Ayw*	26	91 (14.1%)	114 (35.3%)	29 (4.4%)	6
*Cmm*	0	381 (12.8%)	197 (19.0%)	836 (28.0%)	40
*Cms*	63	826 (28.7%)	181 (21.9%)	455 (15.8%)	35
*Cpa*	8	110 (16.3%)	2 (7.5%)	93 (13.8%)	7
*Cpm*	2	43 (9.0%)	7 (4.6%)	79 (16.6%)	8
*Eca*	48	436 (9.9%)	169 (13.5%)	259 (5.9%)	46
*Lxx*	4	612 (30.2%)	92 (13.6%)	211 (10.4%)	47
*MfL*	0	2 (0.3%)	1 (1.4%)	49 (7.2%)	13
*Oyp*	9	118 (15.8%)	99 (28.5%)	25 (3.4%)	7
*Psp*	20	728 (14.7%)	103 (9.3%)	166 (3.3%)	44
*Pss*	19	333 (6.6%)	133 (11.7%)	410 (8.1%)	43
*Pst*	34	766 (14.1%)	174 (10.6%)	209 (3.8%)	44
*Rs*1000	12	503 (14.7%)	200 (20.4%)	150 (4.4%)	40
*Xac*	39	1146 (26.8%)	167 (10.4%)	372 (8.7%)	27
*Xcc*8004	5	1341 (31.4%)	134 (8.4%)	415 (9.7%)	45
*Xcc*33913	7	1022 (24.5%)	131 (8.9%)	349 (8.4%)	45
*Xcc*100	0	790 (17.7%)	91 (5.5%)	432 (9.7%)	29
*Xcv*	10	859 (19.2%)	124 (10.2%)	408 (9.1%)	45
*Xoo*311018	37	1282 (29.6%)	131 (8.3%)	404 (9.3%)	42
*Xoo*10331	6	1586 (38.3%)	152 (11.9%)	470 (11.4%)	40
*Xoo*99A	51	2434 (49.3%)	54 (4.2%)	673 (13.6%)	41
*XfM*12	0	354 (16.8%)	111 (14.4%)	224 (10.5%)	29
*XfM*23	0	324 (15.0%)	83 (12.3%)	734 (34.0%)	29
*Xf*9a5c	70	916 (34.0%)	194 (12.9%)	205 (7.6%)	41
*XfT*	27	459 (22.9%)	114 (15.4%)	370 (18.4%)	41

^a^Full name of all species are listed in Table 1.

^b^HPs indicate hypothetical proteins.

^c^PHX genes indicate predicted highly expressed genes.

**Figure 3:**
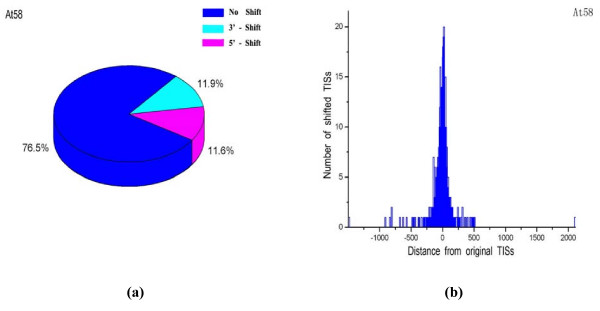
**Statistics of relocated TISs for *At*58**. **(a) **The statistical caky chart for *At*58. Blue regions denote the percentage of the same TISs as the RefSeq annotation. Pink and light blue regions denote the percentage of 5'-shift and 3'-shift from the RefSeq annotation, respectively. **(b) **The histogram of relocated TISs for *At*58. Negative and positive values in *x*-axis indicate the length of 5'-shift and 3'-shift from the RefSeq annotation, respectively, and *y*-axis indicates the number of shifted TISs.

Secondly, a large number of TISs were relocated, and the number and percentage for each genome is listed in the third column of Table 2. The relocated TISs are provided in the 'shift' column of the 'basic information' in DIGAP. Positive and negative numbers indicate the 3'-downstream and 5'-upstream shift of the original TISs, respectively. Most corrected TISs are both predicted by ProTISA and GS-Finder, and many of them have 5' conserved domain confirmed (CDC) and high similarity confirmed (HSC) information [13]. In total, 0.3% ~ 49.3% TISs were relocated in different phytopathogen genomes. As an example, Figure 3 (a) and 3 (b) show the statistical caky chart and histogram of relocated TISs in *At*58. It can be observed that 11.6% (11.9%) of TISs are relocated to the 5'-upstream (3'-downstream) region. Furthermore, the distribution pattern of shifted distances is similar to a normal distribution. The statistical caky charts and histograms for other plant pathogens are shown in the 'documents' section of the website http://ibi.hzau.edu.cn/digap/document.php.

Thirdly, using sequence alignment tools BLAST [15], 1.4% ~ 35.3% of the 'hypothetical proteins' were assigned with functions in different phytopathogen genomes (fourth column of Table 2). All the 'hypothetical proteins' assigned with functions are marked in red in the DIGAP. Most of these proteins have high sequence identity and sequence alignment coverage to their homologs with known functions. To further confirm the reliability of the predicted functions, experimentally characterized homologs were searched in Swiss-Prot and PDB. Many PDB homologs have been identified, which possess the same functions as the predicted functions for 'hypothetical proteins'. Furthermore, PubMed references for the predicted functions of hundreds of homologs of 'hypothetical proteins' are listed in DIGAP. Some predicted functions have experimentally characterized Swiss-Prot homologs, which are listed in Table VI of DIGAP 'statistics' section http://ibi.hzau.edu.cn/digap/statistics.php#6. In total, predicted functions have been assigned to 3683 'hypothetical proteins' in these plant pathogens, and 296 of them have PDB homologs. In addition, more than 600 related references of homologs for the predicted functions are listed in DIGAP.

Finally, 54 potential bactericide targets were identified in these phytopathogens, http://ibi.hzau.edu.cn/digap/targets.php, of which 44 potential targets exist commonly in more than half of the plant pathogens with relatively high sequence identity (>30%), and might serve as promising broad-spectrum bactericide targets in plant protection. The other 10 potential targets exist only in a few genomes with low sequence similarity, which might be used as species-specific bactericide targets. 3D structures of 45 potential targets were modeled, most of which have high sequence identity with their templates in PDB. Furthermore, 25 template enzymes can provide the information of active sites and inhibitors, which are highly valuable for new bactericide discovery.

**Figure 4:**
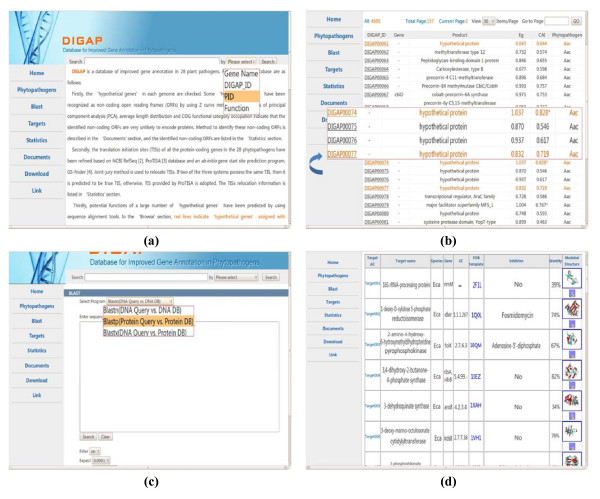
**Web interface of DIGAP**. **(a) **Query interface. **(b) **An example of improved phytopathogen annotation, the 'hypothetical proteins' assigned with functions are marked in red. Users can click DIGAP_ID to obtain the detailed information. **(c) **The BLAST search webpage. **(d) **The potential bactericide targets interface.

DIGAP is supported with a user-friendly designed web interface, so that users can easily get the desired information at any time. Figure 4(a) ~ (d) show some frequently used webpage. As shown in Figure 4(a), users can make a quick search by using gene name, DIGAP_ID, PID and gene function. Figure 4(b) illustrates an example of a phytopathogen annotation, the 'hypothetical proteins' assigned with functions are marked in red in the database. Users can click DIGAP_ID to obtain the detailed annotation information. Figure 4(c) shows the BLAST search webpage. Users can query nucleotide or protein sequences, and the BLAST generates a list of hits which are organized according to the sequence identity between query and object sequences. Figure 4(d) exhibits the potential bactericide targets, which includes the information of PDB template, inhibitor and modeled structure.

## Conclusion

DIGAP is designed to provide improved annotations for the sequenced bacterial phytopathogen genomes, and contains 28 genomes in the current version. With the development of next-generation high-throughput genome sequencing, more bacterial plant pathogen genomes will soon be sequenced, and their improved annotations will be added to DIGAP. The improved annotations will enable a better understanding of lifestyle, metabolism and pathogenicity of these bacterial plant pathogens at molecular level, and will provide valuable resources for controlling phytopathogenic diseases.

## Availability and requirements

The DIGAP database is freely available through the URL: http://ibi.hzau.edu.cn/digap.

All the refined information can be accessed by manual download.

## Authors' contributions

L-LC designed the database, NG and WW established the database. NG, H-FJ, J-WC, BG and LZ collected the data and performed the calculation. All authors analyzed the data. L-LC, S-CZ. and H-YZ wrote the paper. All authors read and approved the final manuscript.

## Supplementary Material

Additional file 1 **Method for recognizing non-coding 'hypothetical ORFs'**. A description of the method for recognizing non-coding 'hypothetical ORFs' Click here for file

Additional file 2 **Methods for calculating *E*(*g*) and CAI indices**. A description of the methods for calculating *E*(*g*) and CAI indices Click here for file
