# Supplementary material for: DIGAP - a Database of Improved Gene Annotation for Phytopathogens
Source: BMC Genomics. 2010 Jan 21;11:54. doi: 10.1186/1471-2164-11-54 (PMC2825234; doi:10.1186/1471-2164-11-54)
Supplement: Additional file 2 — Methods for calculating E(g) and CAI indices. A description of the methods for calculating E(g) and CAI indices [file 1471-2164-11-54-S2.DOC]

**Additional file 2**

**Methods for calculating *E*(*g*) and CAI indices**

**The *E*(*g*) index**

Let *F* and *G* be two groups of genes, the codon usage difference of *F* relative to *G* is calculated by

$B\left(F|G\right)=\sum_{a} p_{a}(F)\left[\sum_{\left(x,y,z\right)=a} \left|f\left(x,y,z\right)-g\left(x,y,z\right)\right|\right]$ (4)

where $p_{a}(F)$ are the average amino acid frequencies of the genes in *F*. $f(x,y,z)$ and $g(x, y, z)$ are the average codon frequencies for the codon triplet $(x,y,z)$ of each amino acid codon family for gene groups *F* and *G*, respectively. The assessments of Eq. (4) can be made for any two gene groups from the same genome or from different ones.

Let $B(g|G)$ indicate the codon usage difference of the gene *g* relative to the gene group *G*, and *C* is the collection of all genes encoded in the genome. Generally, a gene is PHX if its codon usage is similar as the three gene classes RP, CH and TF. Predicted expression levels with respect to individual standards are based on the following

$E_{RP}(g)=\frac{B(g|C)}{B(g|RP)}$, $E_{CH}(g)=\frac{B(g|C)}{B(g|CH)}$, and $E_{TF}(g)=\frac{B(g|C)}{B(g|TF)}$ (5)

The final expression measure is

$E=E\left(g\right)=\frac{B\left(g|C\right)}{\frac{1}{2}B\left(g|RP\right)+\frac{1}{4}B\left(g|CH\right)+\frac{1}{4}B\left(g|TF\right)}$ (6)

PHX genes must satisfy the following two conditions, the *E*(*g*) exceeds 1.00 and at least two of the three expression ratios $E_{RP}(g)$, $E_{CH}(g)$, and $E_{TF}(g)$ are more than 1.05 [1].

**The CAI index**

The CAI index assigns a parameter, termed `relative adaptiveness' by Sharp and Li, to each of the 61 codons (stop codons excluded) [2]. The relative adaptiveness of a codon is defined as its frequency relative to the most often used synonymous codon. This parameter is computed from a set of highly expressed genes G. It is given by

$w_{aa,i}(G)=\frac{f_{aa,i}(G)}{f_{aa,\max}(G)}$ (7)

where *f_aa,i_* is the frequency of codon *i* (which encodes amino acid *aa*), and *f_aa,max_* the frequency of the codon most often used for encoding amino acid *aa* in a set of highly expressed genes *G*. The relative adaptiveness parameter *w_aa,i_* ranges from 0 to 1, with 0 indicating that a codon is not present at all in *G*, and 1, a codon that occurs most often in G for a given amino acid.

The CAI of a gene *g* is simply the geometric average of the relative adaptiveness of all codons in a gene sequence

$CAI_{g}=\prod_{i=1}^{N} w{}_{i}^{1/N}$ (8)

Here, *w_i_* is the relative adaptiveness of the *i*th codon in a gene with N codons. This formula can be transformed into

$CAI_{g}=\prod_{k=1}^{61} w{}_{k}^{X_{k,g}}$ (9)

where *w_k_* now represents the relative adaptiveness of the *k*th out of the 61 codons in the genetic code (excluding stop codons); *X_k,g_* is the fraction of codon *k* among the total number of codons in gene *g*

$X_{k,g}=\frac{C_{k,g}}{\sum_{i=1}^{61} C_{i,g}}$ (10)

where *C_k,g_* is the number of times codon *k* appears in gene *g*. Note that *w_k_ = w_k_(G)* in equation (9) is dependent on the set of highly expressed genes *G*.

Like the relative adaptiveness, the CAI also ranges from 0 to 1. Higher CAI values indicate genes that are more likely to be highly expressed [2, 3].

**References**

1. Karlin,S., Mrázek,J. and Campbell,A.M. (1998) Codon usages in different gene classes of the Escherichia coli genome. *Mol. Microbiol*., **29**, 1341-1355.
2. Sharp,P.M. and Li,W.H. (1987) The codon Adaptation Index--a measure of directional synonymous codon usage bias, and its potential applications. *Nucleic Acids Res*., **15**, 1281-1295.
3. Jansen,R., Bussemaker,H.J. and Gerstein,M. (2003) Revisiting the codon adaptation index from a whole-genome perspective: analyzing the relationship between gene expression and codon occurrence in yeast using a variety of models. *Nucleic Acids Res*., **31**, 2242-2251.
